# Merkel Cell Carcinoma in Immunosuppressed Patients

**DOI:** 10.3390/cancers6031328

**Published:** 2014-06-27

**Authors:** Janice E. Ma, Jerry D. Brewer

**Affiliations:** 1 Mayo Clinic College of Medicine, Mayo Clinic, 200 First St SW, Rochester, MN 55905, USA; E-Mail: ma.janice@mayo.edu; 2 Department of Dermatology, Mayo Clinic, 200 First St SW, Rochester, MN 55905, USA

**Keywords:** immunocompromised patients, immunosuppression, Merkel cell carcinoma

## Abstract

Merkel cell carcinoma (MCC) is a rare and aggressive cutaneous malignancy. The infectivity of Merkel cell polyomavirus (MCPyV), an apparent agent in MCC development, may be exacerbated with impaired immune responses. This paper reviews relevant data regarding the role of immunosuppression in the development of MCC and describes modes of immunodeficient states. Because of the inherently low incidence rate of MCC, several case studies and series are also briefly mentioned to provide a more comprehensive summary of MCC in the setting of immunosuppression. We describe immunosuppressed patients who have experienced excessive UV radiation, organ transplantation, human immunodeficiency virus infection/AIDS, autoimmune diseases, and lymphoproliferative disorders. Iatrogenic forms of immunosuppression are also highlighted. Studies that quantify risks consistently report that individuals with a history of solid organ transplantation, autoimmune diseases, AIDS, and/or lymphoproliferative diseases have a significantly elevated risk of developing MCC. Overall, immunocompromised patients also appear to have an early onset and more aggressive course of MCC, with poorer outcomes. Recommendations for multidisciplinary approaches are proposed to effectively prevent and manage MCC in these patients.

## 1. Merkel Cell Carcinoma: Introduction

Merkel cell carcinoma (MCC) is a rare and highly aggressive neuroendocrine cancer of the skin. MCC is thought to originate from the nerve-associated Merkel cell touch receptors, which are in the layer of basal cells at the deepest portion of the epidermis [1]. With a 33% mortality rate, MCC is deadlier than other more common forms of skin cancer [2]. MCC is associated with local recurrence, regional metastasis, and distant metastasis to the brain, bone, liver, lung, and heart [3]; it is the second most common cause of skin cancer death after melanoma, with an estimated cause-specific death rate of 0.6 per 100,000 persons (in 2006) [4,5]. In addition, the death rate of MCC has been rising rapidly—in the past two decades, deaths due to MCC have increased more than 3-fold [6]. In 1992, cytokeratin 20 (CK-20) was identified as a key diagnostic immunohistochemical marker for MCC [7,8]. Before diagnosis using CK-20, arduous and intensive electron microscopy was required to accurately distinguish MCC from other carcinomas [9]. Thus, many earlier cases of MCC plausibly were misdiagnosed or unreported. In conjunction with the increasingly aging population, the discovery of CK-20 may partially explain the surge in MCC cases in recent decades [10]. 

The primary risk factors for MCC include older age, white race, exposure to UV radiation, and immunosuppression [11,12,13]. With an average age at onset of approximately 74 to 76 years, MCC in the United States is more common among elderly white men with decreased immune function [14]. The higher incidence in non-Hispanic whites may be attributable to their relatively lower amounts of protective skin pigment [5]. Notably, a Danish study reported a higher MCC incidence rate among females, reflecting the higher proportion of women in these older populations [15]. The high mortality rate of MCC may be partially explained by the advanced age at onset in combination with decreased immune function of the elderly as a function of aging [16,17].

In this paper, we review the role of immunosuppression in the development of MCC. The data highlighted in this report include research on MCC in the immunosuppressed setting from the United States, Australia, and European countries. Given that most of the MCC literature is limited to white patients, future research on this tumor in other populations is warranted. In a retrospective study of MCC from mainland China, none of the 22 patients identified with MCC had human immunodeficiency virus (HIV)/AIDS, lymphoma, or any other common forms of immunosuppression [18]. Authors from areas with low inherent incidence rates of nonmelanoma skin cancers (e.g., Korea, Japan) also report few cases of MCC, making it difficult to ascertain relationships in these areas of the world [19,20,21]. 

Most reports of MCC in the setting of immunosuppression are relatively brief compared with well-described skin cancer counterparts such as malignant melanoma. Both MCC and malignant melanoma share risk factors (e.g., UV exposure, immunosuppression), but melanoma has a higher incidence rate and MCC generally has a worse overall prognosis [14,15,22]. A large population-based study found a significant 3-fold increased risk (95% CI, 1.74–4.95) of MCC development in patients with malignant melanoma [22]. The immunosuppressed microenvironment of malignant melanoma may be conducive for development of secondary malignancies, including MCC [23].

### 1.1. MCC and Immunosuppression

Immunosuppressed individuals constitute approximately 10% of the MCC patient population [24]. Immunosuppression increases the risk of MCC and appears to be associated with a worse prognosis [25]. Systemic immune suppression is associated with poorer MCC survival, independent of stage at presentation (*i.e*., local, regional, distant), with a 3-year MCC-specific survival nearly half that of nonimmunosuppressed individuals [25]. Likewise, other research has indicated that MCC survival is linked with strong intratumoral immune responses [26]. Reports have described MCC onset at an earlier age in immunosuppressed patients, particularly organ transplant recipients (OTRs) and patients with HIV/AIDS [13,27]. Another study did not report a statistically significant difference in age of MCC onset in immunosuppressed patients, likely because the majority of these individuals had chronic lymphocytic leukemia, a cancer typically presented in older adults. [24]. Immunosuppression-related risk factors for MCC development include UV-induced immunosuppression, organ transplantation, HIV/AIDS, autoimmune diseases, and lymphoproliferative disorders (LPDs) [13,22,28,29]. 

### 1.2. Merkel Cell Polyomavirus

The Merkel cell polyomavirus (MCPyV) was discovered in 2008, suggesting a link between MCC and immune suppression. Like all polyomaviridae, MCPyV is a small, circular, nonenveloped, double-stranded DNA virus that integrates into the tumor genome in a clonal manner. Although most individuals are naturally exposed to MCPyV, very few have MCC; therefore, other factors such as an immunosuppressed state likely contribute to viral integration, mutagenesis, and carcinogenesis [30]. Other tumors that have viral origins include Kaposi sarcoma and Burkitt lymphoma; these also have a higher incidence in immunodeficient patients [31]. 

The large T-antigen expressed in MCC tumors in the truncated form inhibits retinoblastoma tumor suppressor genes and promotes cell division. Large T-antigen mutations are characteristic of MCPyV-positive MCC tumor cells [32]. MCPyV is present in 75% to 80% of MCCs, whereas it was identified in 16% of control tissue samples [33,34]. MCPyV has been detected in other cutaneous tumors, but in some reports, the evidence indicating the presence of MCPyV was mixed [33,34,35,36,37]. 

MCC development appears to have two distinct etiologic pathways: MCPyV mediated and non-MCPyV mediated [38,39]. Analyses of primary and metastatic MCC tumors show that an infiltration by T cells (CD8^+^, CD4^+^, and CD3^+^) and increased immune response transcripts are associated with tumor regression and improved prognosis [25,40,41]. The high intratumoral T-cell counts are even associated with better prognosis in MCPyV-negative MCC [42]. The effect of MCPyV on the clinical course of patients with MCC is uncertain. Two studies have shown that individuals with MCPyV-positive tumors have a better prognosis than those with MCPyV-negative tumors [39,43]. Accordingly, MCPyV-negative MCCs specifically harbor mutations in the tumor suppressor gene TP53 that are linked to worse outcomes in cancers because of resistance to cancer therapies [44,45]. In contrast, other studies have observed no statistically significant survival differences between patients with MCPyV-positive and MCPyV-negative tumors [46,47]. These conflicting findings may be explained by differences in geographical origin and timing of patient cohort collection, chance, or technical factors such as different MCPyV assays. 

MCC develops despite the presence of both humoral and cellular responses against MCPyV infection. Antibodies against MCPyV viral capsid proteins, particularly immunoglobulin G, are detected in up to 80% of healthy adults (>50 years old) [48]. MCC tumors notably do not express viral capsid proteins. Some have proposed that immunocompromised patients with MCC have higher antibody titers and viral loads [49]. Humoral immune responses may promote antitumor activity, although its contribution is unproven [26]. Meanwhile, cellular responses target MCPyV through virus-reactive CD8^+^ and CD4^+^ T cells [50]. Comparative studies that have investigated MCPyV and other polyomaviruses in immunosuppressed individuals such as OTRs have found relatively low levels of MCPyV and were inconclusive [31,51]. Better understanding of the pathogenesis of MCPyV and its interaction with the immune response could enable more effective treatments for MCC. 

## 2. MCC and UV-Induced Immunosuppression

UV radiation is a known risk factor for many skin cancers [52]. It can cause mutagenic, carcinogenic, and immunosuppressive effects [53,54]. UV radiation promotes DNA damage, induces the immunosuppressive cytokines interleukin-1 and tumor necrosis factor α, and generates reactive oxygen species; the decrease in DNA repair and subsequent immunosuppression contribute to carcinogenesis [55]. Solar radiation is a major risk factor for MCC. Accordingly, MCC typically develops in sun-exposed skin surfaces, notably the head and neck, followed by the extremities [4,5]. 

UV-B rays are less prevalent than UV-A rays, but they are much more intense and destructive [56]. UV-B induces mutations in the tumor suppressor p53 and Ha-ras genes, which increase the risk of cancer [57]. The UV-B index, the quantitative geographic measurement of radiation exposure, is positively associated with MCC incidence across US cities [58]. This observation is supported by a study showing greater prevalence of non-MCPyV-mediated MCC in Australia than in North America [59], possible because of the increased sun exposure in Australia. 

In 2010, Mogha *et al.* [60] demonstrated a molecular link between MCC and sun exposure. The mRNA transcript of MCPyV small t antigen had a dose-dependent increase after UV radiation (in the form of solar-simulated radiation). MCC patients with MCPyV VP1 antibody titers >10,000 had a significantly better prognosis than controls [61]. In addition, progression-free survival was observed in groups with higher antibody levels. Further, UV-induced mutations (*i.e*., pyrimidine dimers) impair helicase and prevent replication, which in turn could promote the survival of MCPyV. Specifically, there is a high frequency of pyrimidine dimer substitutions in large T-antigen mutations [32].

UV-A has also been reported to induce MCC. This long-wavelength UV corresponds to deeper penetrance beyond the epidermis into the dermis and is a significant contributor to UV-induced immunosuppression [62]. In a nationwide US study (1975–1998), two of 1,380 patients with psoriasis treated with methoxsalen (psoralen) and UV-A photochemotherapy had MCC develop more than 20 years later [63]. Calzavara-Pinton *et al.* [64] also identified two immunosuppressed patients with MCC development after high-dose UV-A1 (320–400 nm) phototherapy. On a molecular level, analyses of MCC cell lines showed oxidative damage induced by environmental factors, especially UV-A, which resulted in chromosomal imbalances [57].

## 3. MCC in Solid OTRs

OTRs are a well-documented subset of immunosuppressed patients. They often receive medications or therapies that result in immunodeficiency to prevent organ rejection, but long-term immunosuppressant regimens may promote carcinogenesis [65,66]. Skin cancer is the most common posttransplant malignancy, affecting at least 50% of OTRs [55,67]. In addition, OTRs with low CD4^+^ counts have more aggressive skin cancers, including MCC [68,69]. 

### 3.1. Posttransplant Incidence of de Novo MCC

In the literature, MCC is mostly linked with kidney, liver, and heart transplantation. On average, MCC tumors arise 7.6 years after transplantation (range, 5–286 months) [70]. MCC incidence in OTR is higher in males, reflecting the 2:1 ratio of male:female heart transplant recipients (HTRs) and liver transplant recipients (LTRs) [27,71]. Epidemiologic analyses have suggested that MCC arises in OTRs at a mean age of 53 years, significantly earlier than the typical ≥70-year age range [27,71,72,73]. Case reports have described atypical localization of MCC in OTRs, *i.e*., in areas without sun exposure such as the buttocks and gluteal regions [74,75,76]. 

Epidemiologic studies of OTRs are unstandardized and present different risks in relation to MCC development. Table 1 summarizes the risks of MCC occurring in OTRs as outlined in single-center, multicenter, or registry analyses. Of the seven studies outlined, three registry analyses conducted in different countries quantified risk. All three registry analyses showed a significant risk of posttransplant MCC, including an Australian study that reported a 103-fold increased risk of MCC for HTRs [27,58,77]. These epidemiologic studies suggest that regardless of age group, OTRs appear to have significantly higher risk of MCC development, even when compared with other cancers [77,78]. 

**Table 1:** Summary of relative risks of MCC occurring after solid organ transplantation.

Reference	Study Type (Country)	Organ	No. of patients	No. of Patients With MCC	SIR or OR 95% confidence interval ^a^
Buell *et al.* 2002 [71]	Multicenter (international) study (IPITTR)	Kidney, heart, liver	≥15,000 OTR	45	--
Baccarani *et al*. 2006 [79]	Single-center study (Italy)	Liver	582 OTR (202 LTR)	1	--
Koljonen *et al.* 2009 [27]	Registry analysis (Finland)	Kidney	4200 RTR	3	SIR = **66** 95% CI = 14–194
Basic-Jukic *et al.* 2010 [80]	Single-center study (Croatia)	Kidney	1232 RTR	1	--
Kalinova *et al.* 2010 [81]	Single-center study (Czech Republic)	Kidney	603 RTR	1	--
Lanoy *et al.* 2010 [58]	Registry analysis (USA-SEER) ≥65 years old	Kidney, heart, lung, liver	1286 OTR	11	OR = **4.95** 95% CI = 2.62–9.34
Na *et al.* 2013 [77]	Registry analysis (Australia)	Heart	1,518 HTR	17	SIR = **103** 95% CI = 60.4–166

Abbreviations: CI, confidence interval; HTR, heart transplant recipients; IPITTR, Israel Penn International Transplant Tumor Registry; LTR, liver transplant recipients; MCC, Merkel cell carcinoma; OR, odds ratio; OTR, organ transplant recipients; RTR, renal transplant recipients; recipients; SEER, Surveillance, Epidemiology, and End Results; SIR, standardized incidence ratio. ^a^ Statistically significant risk values are shown in **boldface** type.

It appears that the organ type does not affect the incidence of posttransplant MCC. For instance, a retrospective single-center study in Italy identified one case of MCC arising in LTRs, but none in renal transplant recipients (RTRs) [79]. Meanwhile, retrospective analyses in Croatia and Czech Republic described MCC arising in RTRs [80,81]. A retrospective registry analysis of 45 *de novo* MCC cases reported to international centers in the Israel Penn International Transplant Tumor Registry (IPITTR) found that MCC cases occur most commonly in kidney (*n =* 40), followed by heart (*n =* 3) and liver transplant (*n =* 2) recipients [71]. Instead of the organ transplant itself, these variations in MCC development may be attributable to the different immunosuppressive regimens of OTRs (see Section 3.3). 

### 3.2. Clinical Course of MCC in OTRs

Studies have been conducted on the incidence of posttransplant MCC, but outcome data are limited. Nevertheless, studies have observed high mortality rates in this context [68,71]. A study that described three RTRs with subsequent MCC reported death from metastatic MCC within 0.5 to 2.1 years for all patients [27]. Based on a review of all *de novo* MCC cases reported internationally to the IPITTR, Buell *et al.* [71] described 45 patients presenting with 48 cases of MCC. Of these OTRs, 27 (60%) died of MCC, which suggests a prognosis worse than the 33% mortality rate of non-OTR patients with MCC. MCC also had a 31% recurrence rate in OTRs. OTRs with subsequent development of MCC may have a more aggressive disease course with worse overall survival rates; however, larger studies are required for a better understanding of this unique patient population. 

### 3.3. Immunosuppressive Treatments in OTRs

After transplantation, the medications administered to OTRs to prevent graft rejection result in iatrogenic immunosuppression [82]. However, the effects of specific immunosuppressive medications on MCC development in OTRs remain unknown. Calcineurin inhibitors cause a 200-fold increase in skin cancer risk and have tumorigenic effects by interfering with DNA repair [83]. Azathioprine, a purine analogue, also is linked to a higher risk of nonmelanoma skin cancers [84]. Case reports have described patients with metastatic MCC regression after withdrawal of azathioprine and cyclosporine, although remission did not reach a year in these cases [85,86]. 

Some have suggested modifying or decreasing immunosuppressive regimens as a way to improve the clinical course of post-transplant skin cancer; however, information on this strategy is very limited with regard to MCC, and the potential risk of skin cancer must be balanced against the possible risk of organ rejection. Recent studies advocate use of rapamycin agents (e.g., sirolimus, everolimus), which inhibit the mammalian target of the rapamycin (mTOR) pathway, over the use of calcineurin inhibitors [87]. This change may help manage the course of skin cancers by reducing incidence and improving survival in general [88]. 

## 4. MCC and Lymphoproliferative Diseases

B-cell LPDs, including leukemia, multiple myeloma, and lymphoma, are neoplasms of the blood involving abnormal proliferation of lymphocytes. Immunosuppression is a risk factor for both MCC and LPDs, especially non-Hodgkin lymphoma (NHL), chronic lymphocytic leukemia (CLL), and multiple myeloma [89]. Individuals with LPDs have greater risk of a second neoplasm, especially skin cancer [90]. Interestingly, lymphoma is the most common secondary malignancy in patients with existing skin cancers. The association between lymphomas and nonmelanoma skin cancers (basal and squamous cell carcinomas) is well documented; however, its relationship with MCC has not been described until more recently [91,92,93]. 

### 4.1. Molecular Mechanisms of LPDs and MCC Development

CLL, a low-grade B-cell malignancy, is a subtype of NHL. Individuals with CLL have weakened humoral and cell-mediated immune responses that may promote MCPyV infection or MCC development (or both) [94]. MCPyV may be involved in CLL pathogenesis, or possibly the immunosuppressed state merely promotes reactivation and proliferation of the virus [95]. The latter is supported by a report describing a patient with follicular lymphoma who had MCC onset soon after treatment with the immunosuppressive agents fludarabine and rituximab [96]. T-cell-suppressive therapies are often used in lymphoma treatment, which further enhances the immunodeficient state that favors MCPyV invasion and possibly the subsequent development of MCC [72,97]. Moreover, studies have shown that immunosuppressive therapies such as purine analogs and monoclonal antibodies for treatment of LPDs increases risk of MCC development [98]. 

In contrast, studies have reported low levels of MCPyV DNA in about 30% of patients with CLL, which may suggest a viral contribution in the development of MCC malignancy in this subset of immunosuppressed patients [95,99]. In accordance with this finding, Koljonen *et al.* [100] also identified MCPyV DNA in five of five samples of CLL-MCC patients. MCPyV seroprevalence was higher in patients with lymphoma than the general population, although this difference was not statistically significant [101]. 

Multiple myeloma impairs the humoral immune response, which makes those affected more susceptible to infections [102]. Several cases of MCC arising in patients with a history of multiple myeloma have been documented [103,104]. Exogenous immunosuppression or history of hematopoietic disease may promote MCC metastases to nonclassical regions such as the bone marrow [105,106,107,108,109]. A recent case report described a patient with metastatic MCC in the bone marrow not involving T-cell suppression, thereby implicating B-cell depression [104]. MCC also was reported in non–B-cell derived myeloproliferative disorders (hairy cell leukemia and T-cell lymphoma), although molecular mechanisms have not been detailed [110].

### 4.2. LPDs and MCC Epidemiology

Analyses of cancer registries yield mixed findings. Registry analyses quantifying the risk association between MCC and LPDs from various countries are summarized in Table 2 and Table 3. Registry analyses of the relative risks of LPDs occurring after MCC have been conflicting: some studies found a significantly elevated risk and others found an insignificant difference. Studies have reported that patients with MCC have a 0.7- to 4.5-fold increased risk of NHL and a 2.7- to 48-fold increased risk of CLL [15,22,24,100,111]. Meanwhile, published studies have found a consistent, significant 3.4- to 10.2-fold elevated risk of MCC developing after LPDs [15,22]. In spite of the wide range of findings across databases of different nations, a relationship clearly exists between MCC and LPDs, especially NHL and CLL. 

**Table 2:** Summary of relative risks of LPDs occurring after MCC.

Reference	Country (Years of Registry)	Type of LPD	No. of MCC Patients	No. (%) of LPDs After MCC Diagnosis	SIR ^a^	95% CI
Howard *et al.* 2006 [22]	USA (1986–2002)	NHL	1306	10 (0.77%)	**2.56**	1.23–4.71
Koljonen *et al.* 2010 [100]	Finland (1979–2006)	NHL	172	2 (1.2%)	4.52	0.55–16.3
Bzhalava *et al.* 2012 [111]	Denmark, Norway, Sweden (1980–2007)	NHL	756	1 (0.13%)	0.7	0.1–4.97
Howard *et al.* 2006 [22]	USA (1986–2002)	CLL	1306	3 (0.23%)	2.72	0.55–7.94
Heath *et al.* 2008 [24]	USA (1980–2007)	CLL	195	8 (4.1%)	**48** (≤70 y old) **34** (≥70 y old)	--
Koljonen *et al.* 2010 [100]	Finland (1979–2006)	CLL	172	2 (1.2%)	**17.9**	2.16–64.6
Kaae *et al.* 2010 [15]	Denmark (1978–2006)	CLL	185	2 (1.1%)	**11**	2.7–43.8
Tadmor *et al.* 2012 [110]	Israel (1989–2010)	45% CLL 29% NHL 6% other	335	M: 4 F: 3 *N =* 7 (2.1%)	M: **3.57** F: **2.94**	M:0.07–7.07 F: 0.00–6.27

Abbreviations: CLL, chronic lymphocytic leukemia; F, female; LPD, lymphoproliferative disorder; M, male; MCC, Merkel cell carcinoma; NHL, non-Hodgkin lymphoma; SIR, standardized incidence ratio; USA, United States of America. ^a^ Statistically significant risk values are shown in **boldface** type.

**Table 3:** Summary of relative risks and mortality risks of MCC occurring after LPDs.

Reference	Country (Years of Registry)	Type of LPD	No. of Patients	No. of MCC After LPDs	SIR or SMR ^a,b^	95% CI
**Relative Risks**						
Howard *et al.* 2006 [22]	USA-SEER (1986–2002)	NHL	81,743	16	**3.37**	1.93–5.47
Kaae *et al.* 2010 [15]	Denmark (1978–2006)	NHL	185	3	**7.3**	2.3–22.8
Howard *et al.* 2006 [22]	USA-SEER (1986–2002)	CLL	17,315	14	**6.89**	3.77–11.57
Kaae *et al.* 2010 [15]	Denmark (1978–2006)	CLL	185	3	**10.2**	3.2–31.9
Howard *et al.* 2006 [22]	USA-SEER (1986–2002)	Multiple myeloma	23,949	4	**3.70**	1.01–9.47
**Mortality Risks**						
Brewer *et al.* 2012 [112]	USA-SEER (1986–2002)	CLL	3613	48	**3.07**	2.20–4.27
Brewer *et al.* 2012 [112]	USA-SEER (1986–2002)	NHL	3613	42	1.85	1.24–2.78

Abbreviations: CLL, chronic lymphocytic leukemia; LPD, lymphoproliferative disorder; MCC, Merkel cell carcinoma; NHL, non-Hodgkin lymphoma; SEER, Surveillance, Epidemiology, and End Results; SIR, standardized incidence ratio; SMR= standardized mortality ratio. ^a^ Statistically significant risk values are shown in **boldface** type; ^b^ SIR for relative risks; SMR for mortality risks.

### 4.3. Prognosis of LPDs in MCC Patients

Literature is limited regarding the prognosis of patients with lymphoma and MCC. A review of seven patients with MCC and a history of CLL showed higher recurrence and metastasis of MCC [113]. A poorer prognosis in these patients was also suggested, but the association was only recently demonstrated in epidemiologic data. In a population-based study of 335 MCC cases in Israel, researchers showed that the presence of hematologic malignancies, both before and after MCC diagnosis, was associated with a significantly higher MCC-specific mortality rate (relative risk, 1.68; *p* < 0.001) [110]. 

Consistent with these data, Brewer *et al.* [112] published the first population-based study analyzing the outcomes of NHL/CLL-MCC patients. Of the 3,613 MCC patients who were identified in the Surveillance, Epidemiology, and End Results (SEER) national database, 90 had a history of lymphoma. Patients with a history of CLL or NHL and subsequent development of MCC had a significantly lower overall survival rate. Compared with MCC patients without CLL or NHL, MCC patients with a history of CLL had significantly worse overall survival (standardized mortality ratio [SMR], 3.1; 95% CI, 2.2–4.3), whereas patients with a history of NHL had an SMR of 1.9 (95% CI, 1.3–2.8). Additionally, this study showed that MCC cause-specific survival was worse for patients with a history of CLL but not NHL. Patients with a history of CLL were nearly 4-fold more likely to die of metastatic MCC (SMR, 3.8; 95% CI, 2.5–5.9), whereas patients with a history of NHL were not significantly different from expected (SMR, 0.9; 95% CI, 0.4–2.1). Results from this study are summarized in Table 3.

Whether there is a correlation between the duration from diagnosis of CLL to onset of MCC and the patient’s prognosis is subject to debate. Several case reports [91,114,115] proposed that a shorter interval was associated with better prognosis, whereas another case report described a rapid, fatal outcome [116]. 

## 5. MCC in Patients with HIV Infection/AIDS

### 5.1. Molecular Mechanisms of HIV/AIDS and MCPyV

The pathogenesis of MCC in HIV-infected patients is not well elucidated. HIV infection destroys CD4^+^ T cells and impairs the immune system’s ability to fight infection. To date, no relationship has been identified between CD4^+^ cell counts and MCC cause-specific survival in HIV-infected patients [117]. However, there is a higher risk of MCPyV-induced MCC in immunosuppressed patients [118]; a recent report compared HIV-infected men with or without severe immunosuppression and showed significantly higher cutaneous MCPyV DNA loads in those with severe immunosuppression [119]. Another recent study detected significantly higher levels of MCPyV DNA in sera of immunocompromised HIV-1–positive patients who had not received highly active antiretroviral therapy (HAART) compared with HIV-1–negative hosts (*p* < 0.01) [120]. 

### 5.2. HIV/AIDS and MCC Co-Occurrence

The course of MCC in patients with HIV/AIDS can be aggressive and associated with poor survival [121,122,123,124]. On average, MCC arises two decades earlier in patients with HIV/AIDS compared with immunocompetent individuals [28]. A US-based study indicated the majority of HIV/AIDS-MCC patients are men [125]. In addition, MCC tumors in patients with HIV/AIDS do not arise in the typical areas (e.g., head and neck), indicating that UV exposure may not be a primary causative factor; in one review, 35% of the HIV-MCC patients had MCC tumors develop on the buttocks [28]. Furthermore, in HIV-infected patients, primary MCC tumors were even observed in noncutaneous sites such as the intraparotid lymph nodes, although those cases could represent stage III MCC with an unknown primary tumor [126]. 

Epidemiologic studies of MCC predisposition in HIV-infected individuals highlight distinct findings across different age groups. A 2002 study of the US registry showed that HIV-infected individuals had a 13.4-fold higher risk of MCC development compared with the general population [13]. This HIV-MCC population consisted of younger adults, with no individuals older than 65 years. Another US registry analysis of younger adults (median age, 39 years) also showed a greatly increased risk of MCC in patients with AIDS (standardized incidence ratio [SIR], 11; 95% CI, 6.3–17) [125]. Meanwhile, a recent case-control study showed that the risk of MCC in HIV-positive older adults (≥65 years) in the Medicare dataset was not significantly increased (OR, 2.30; *p* = 0.07) compared with HIV patients in the control group [58]. 

The use of HAART has improved survival of HIV-infected patients; however, no clear consensus exists on the effect of HAART on MCC development and behavior in patients with HIV/AIDS [122]. Case reports have described sustained remission of metastatic MCC in HIV-positive patients with HAART and interleukin-2 treatment [127,128]. However, by prolonging the lives of patients with HIV/AIDS, HAART may also result in more cases of MCC [13]. 

## 6. MCC and Autoimmune Diseases

Autoimmune disorders impair the natural immune response, creating an environment that makes an affected individual particularly vulnerable to secondary malignancies [129]. Several autoimmune disorders have been linked to an increased incidence of MCC (Table 4). In a Swedish retrospective registry analysis, Hemminki *et al.* [11] reported that the risk of MCC was significantly increased after ankylosing spondylitis (SIR, 15.62; 95% CI, 2.95–46.25), inflammatory bowel disease (SIR, 4.02; 95% CI, 1.82–7.66), and Crohn disease (SIR, 4.38; 95% CI, 1.38–10.31). Meanwhile, the risk of MCC was not significantly different after rheumatoid arthritis (SIR, 2.42; 95% CI, 0.96–5.01). Other studies observed increased risk of MCC in patients with rheumatoid arthritis, which was perhaps at least partially attributable to immunosuppression after chronic use of systemic corticosteroids [12,73]. The use of tumor necrosis factor inhibitors in the treatment of systemic autoimmune diseases may further exacerbate immunosuppression [97]. MCC has been described after the administration of the anti-tumor necrosis factor therapy rituximab [130]. 

The majority of information on autoimmune disorders and MCC is scattered among case reports, which makes it difficult to establish associations [131,132]. However, MCC development has been documented in patients with autoimmune hepatitis, systemic lupus erythematosus, Behçet syndrome, and chronic sarcoidosis and recieving immunosuppressive medications [131,133,134,135,136]. Paraneoplastic autoimmune syndromes, including Lambert-Eaton myasthenic syndrome, have also been linked to MCC, although a relationship with these syndromes and the clinical outcome of MCC is not well established [137]. 

**Table 4:** Summary of relative risks of MCC occurring after various forms of immunosuppression.

Reference	Study Type (Country)	Mode of Immunosuppression	Findings ^a^
Lanoy *et al.* 2010 [12]	Registry analysis. (USA)	Autoimmune disease (RA)	79/1977 MCC patients had RA develop Odds ratio, **1.39** (95% CI, 1.10–1.75)
Cirillo *et al.* 2012 [71]	Single-center analysis. (Italy)	Autoimmune disease (RA)	3/48 patients with RA had MCC develop during immunosuppressant corticosteroid treatment *Statistical significance not determined*
Hemminki *et al*. 2012 [11]	Registry analysis (Sweden)	Autoimmune diseases (AS, IBS, CD, RA)	3/112,541 patients with AS had MCC (SIR, **15.62**; 95% CI, 2.95–46.25) 9/68,915 patients with IBS had MCC (SIR, **4.02**; 95% CI, 1.82–7.66) 5/35,422 patients with CD had MCC (SIR, **4.38**; 95% CI, 1.38–10.31) 7/71,963 patients with RA had MCC (SIR, 2.42; 95% CI, 0.96–5.01).
Engels *et al*. 2002 [13]	Registry analysis (USA)	AIDS	6/30,9365 patients with AIDS had MCC (relative risk, **13.4**; 95% CI, 4.9–29.1)
Lanoy *et al.* 2009 [125]	Registry analysis (USA)	AIDS	17/497,142 male patients with AIDS had MCC (SIR, **11**; 95% CI, 6.3–17)
Lunder and Stern. 1998 [63]	Multicenter analysis (USA)	UV-A phototherapy + psoralen	3/1380 (0.2%) patients with psoriasis receiving UV-A + psoralen had MCC develop ~**100**-fold increase
Calzavara-Pinton *et al.* 2010 [64]	Retrospective analysis (Italy)	UV-A phototherapy	2 immunosuppressed patients had MCC develop after UV-A1 phototherapy treatment *Statistical significance not determined*
Sahi *et al*. 2012 [29]	Registry analysis, 1994–2009 (Finland)	Statins (HMG-CoA-reductase inhibitors)	50/454,935 statin users had MCC develop Age ≤60 y: SIR, 3.16; 95% CI, 0.65–9.23 Age 60–74 y: SIR, **1.94**; 95% CI, 1.23–2.90 Age ≥75 y: SIR, **0.89**; 95% CI, 0.67–0.92
Howard *et al.* 2006 [22]	Registry analysis, 1986–2002 (USA)	Malignant melanoma	16/70,604 patients with melanoma had MCC (SIR, **3.05**; 95% CI, 1.74–4.95)

Abbreviations: AS, ankylosing spondylitis; CD, Crohn disease; IBS, inflammatory bowel syndrome; MCC, Merkel cell carcinoma; RA, rheumatoid arthritis; SIR, standardized incidence ratio; USA, United States of America. ^a^ Statistically significant risk values are shown in **boldface** type.

## 7. Recommendations

Current treatment of MCC is assessed on the basis of the clinical stage of the tumor at presentation [138]. Biopsies, including the low-risk sentinel lymph node biopsy and CT/PET scans are used as diagnostic tests to assess the stage of MCC. While sentinel lymph node biopsy is a reliable staging procedure in many cancers including melanoma, its value in MCC patients remains unclear [139]. Conventional routes of MCC treatment include surgery, radiotherapy, and chemotherapy. Surgical excision is the first-line treatment for localized MCC, often in combination with radiotherapy or chemotherapy (or both) for regional or distant metastases [140,141]. The effect of chemotherapy on the outcome of MCC is controversial; some evidence even suggests that certain forms of chemotherapy may be detrimental [142]. Autologous peripheral blood stem cell transplantation has also been proposed as a mode of treatment, though its effects on MCC are contradictory. One case report achieved a 6-month complete remission of metastasized MCC using a combination of autologous peripheral blood stem cell transplantation and high-dose chemotherapy [143]. Meanwhile, another case report found an onset of fatal, metastatic MCC following autologous peripheral blood stem cell transplantation for non-Hodgkin’s lymphoma [144]. Further research needs to be conducted in order to determine the effect of stem cell transplantation on MCC. 

Multidisciplinary management is key in treating all patients with MCC, especially those with immunosuppression. Annual skin surveillance examinations should be implemented, particularly for patients with solid organ transplantation, HIV/AIDS, a history of LPDs, or other iatrogenic forms of immunosuppression. A lower threshold for biopsy of new or changing skin lesions, especially those in sun-exposed regions, is warranted [141]. Furthermore, education and awareness of MCC is crucial for both clinicians and at-risk individuals. Clinicians and immunodeficient individuals may also benefit from remembering the acronym AEIOU: asymptomatic/lack of tenderness, expanding rapidly (<3 months), immunosuppression, older than 50 years, and UV-exposed site location. A study of 195 patients showed that 89% met at least three of the AEIOU criteria, 52% met four or more, and 7% met all five [24]. Moreover, it is important for immunosuppressed patients to perform monthly self-examinations, avoid tanning beds, and see a dermatologist annually. UV (sun) protection is essential for immunosuppressed patients and should be part of their daily routine. In addition, vitamin D supplementation may be beneficial, especially for OTRs [72,145]. 

Decreasing immunosuppression by modifying immunosuppressive treatments may be beneficial when deemed safe. The administration of interleukin-2 in combination with HAART to bolster T-cell immunity and prevent metastatic spread of MCC in HIV-infected individuals has been suggested [127]. The use of statins, which are immunosuppressive agents, has also been linked to a 3-fold increased risk of MCC development in individuals younger than 60 years [29,146]. 

Immunotherapy has potential use in MCPyV-positive cases. Proposed novel treatments include the use of immune-regulatory cytokines, adoptive cellular infusions, topical immune modulators, and DNA vaccines [26]. Several studies on the application of antiviral type I interferons and tumor necrosis factor on patients with MCC are promising, but more clinical studies are needed [147,148,149]. More research on MCPyV and its role in MCC development in patients with a history of autoimmune diseases, HIV/AIDS, and organ transplantation would lend insight on MCC pathogenesis and origin. 

## 8. Conclusions

Immunosuppressed patients have greater risk of malignancies, including MCC, and also have increased risk of worse cause-specific survival in the setting of MCC. Long-term immunosuppression appears to increase the risk of MCC development and also results in earlier age at onset, more aggressive course, and a worse prognosis. Most reports of MCC in immunosuppressed patients are brief. The limited numbers of MCC cases in the setting of immunosuppression make associations difficult to ascertain. Dermatologic surveillance is crucial in severely immunosuppressed individuals who have undergone organ transplantation or have a history of LPDs, HIV/AIDS, or autoimmune diseases. Immunosuppressive agents appear to increase susceptibility to MCC development. Better understanding of how MCPyV modulates the immune system will be beneficial for developing effective immunotherapies for MCC. Additionally, better understanding of the pathogenesis, prognosis, and management of MCC is necessary for the future direction and recommendations for treatment, surveillance, and outcomes in immunocompromised patients. 

## Abbreviations

CK-20: cytokeratin 20
CLL: chronic lymphocytic leukemia
HAART: highly active antiretroviral therapy
HIV: human immunodeficiency virus
HTR: heart transplant recipient
IPITTR: Israel Penn International Transplant Tumor Registry
LPD: lymphoproliferative disorder
LTR: liver transplant recipient
MCC: Merkel cell carcinoma
MCPyV: Merkel cell polyomavirus
NHL: non-Hodgkin lymphoma
OTR: organ transplant recipient
RTR: renal transplant recipient
SEER: Surveillance, Epidemiology, and End Results
SIR: standardized incidence ratio
SMR: standardized mortality ratio
